# Multiple crack detection in 3D using a stable XFEM and global optimization

**DOI:** 10.1007/s00466-017-1532-y

**Published:** 2018-02-19

**Authors:** Konstantinos Agathos, Eleni Chatzi, Stéphane P. A. Bordas

**Affiliations:** 1 Institute of Theoretical, Applied and Computational Mechanics, Cardiff University, Cardiff, CF24 3AA UK; 2 Research Unit in Engineering Science, Luxembourg University, 6 rue Richard Coudenhove-Kalergi, 1359 Luxembourg, Luxembourg; 3 Institute of Structural Engineering, ETH Zürich, Stefano-Franscini-Platz 5, 8093 Zurich, Switzerland

**Keywords:** Crack detection, XFEM, Genetic algorithms, CMA-ES

## Abstract

A numerical scheme is proposed for the detection of multiple cracks in three dimensional (3D) structures. The scheme is based on a variant of the extended finite element method (XFEM) and a hybrid optimizer solution. The proposed XFEM variant is particularly well-suited for the simulation of 3D fracture problems, and as such serves as an efficient solution to the so-called forward problem. A set of heuristic optimization algorithms are recombined into a multiscale optimization scheme. The introduced approach proves effective in tackling the complex inverse problem involved, where identification of multiple flaws is sought on the basis of sparse measurements collected near the structural boundary. The potential of the scheme is demonstrated through a set of numerical case studies of varying complexity.

## Introduction

The advent of low-cost and easily deployable sensor technologies, has in recent years sparked a significant rise in the deployment of monitoring technologies for large-scale structural systems [1]. Due to the flexibility of technologies involved, Structural Health Monitoring (SHM) methods are available in various forms, i.e., vibration-based [2] or static monitoring [3], periodic and short-term versus continuous and long-term deployments, visual inspections versus non-destructive evaluation [4, 5]. etc.

Availability of monitoring data may be exploited in a number of tasks pertaining to the life-cycle assessment and management of infrastructure systems including condition and reliability assessment [6], updating/calibration of simulation models [7], prediction of performance and residual life (prognostics) [8], damage identification and fault detection (diagnostics) [9]. The damage detection task is one of particular importance and is often considered as the focus of SHM processes, which may be defined across four levels [10]: (i) detection of damage; (ii) localization of damage; (iii) quantification of the severity and extent of damage; and (iv) estimation of the future performance of the component (or system) as damage accumulates.

While the first tasks of damage detection, and potentially localization, may be often achieved on the basis of data processing alone, the more refined diagnostic levels typically require the combined use of a simulation model for the monitored system. Availability of a system model enables formulation of a so-called inverse problem procedure [11], where the task lies in updating the system’s representation in a way which reveals its current status, and is thereby informative with respect to the nature of the induced damage, e.g. fatigue, cracking or component failure. Availability of monitoring data drives the inverse problem formulation, which aims to minimize the difference between the model prediction and the structural response data acquired via monitoring. This may often be solved by means of optimization methods based on least squares or based on Bayesian analysis [12, 13].

In an optimization setting, monitoring data such as acceleration [14], strain [15], acoustic emission, wave propagation [16], or impedance [17] data essentially establish the target function to be optimized, while structural properties and the characteristics of potential damage (geometry, location, extent of flaw) form the optimization variables. The inverse problem solution calls for multiple analyses of the so-called forward problem, i.e., the simulation of the system given prescribed structural and flaw properties. In this sense, it is evident that the problem may become computationally taxing when forward analysis of complex systems is involved, including analyses in the three dimensional space. Since it is oftentimes desired to perform the diagnostic tasks in the short time that follows an initial indication of damage, the corresponding analysis tools ought to ensure rapid computation.

Within this context, a number of techniques have been proposed in recent literature for cutting down on computation while maintaining estimation accuracy, the majority of which rely on reduced order representations. A first approach pertains to the use of surrogate models [18, 19], which are often data-driven albeit not necessarily linked to first principles (physical) information. A second alternative however, pertains to reduced representations that are founded on the principles of computational mechanics, such as multiscale schemes [20] for composite systems, component mode synthesis methods [21] for structural dynamics problems, or the extended Finite Element Method (XFEM) for linear elastic fracture mechanics [22].

In the case of fracture, a significant challenge faced by reduced order representations lies in the tackling of the *non-linearities* that are associated with the fracture or damage process. These typically require inclusion of a large number of modes for accurately capturing the high-frequency nature of the solution around the damage zone. The latter may in general not be entirely precomputed due to the non-linearities in the damage and fracture processes.

To address this, a number of possibilities exist, mainly relying on updating the reduced space on-the-fly. The interested reader is referred to the work of Kerfriden and coworkers and the publications therein, where Newton–Krylov [23], local–global [24] domain-wise model order reduction [25], and Bayesian approaches [26] are proposed. Those algebraic-based model order reduction techniques may be complemented by multiscale approaches, as in [27], where a scale-selection approach is proposed for determining the optimal model for a given region. Finally, statistical-based approaches have been proposed [28] in order to determine the fracture process zone based on the lack of ability of reduced order models to represent the failure of the system.

In this paper, and motivated by previous works of the authoring team in the two-dimensional domain, we rely on XFEM for solution of the forward problem. XFEM alleviates the need for remeshing [29, 30] for diverse flaw locations and geometries thereby significantly cutting down on the computational toll of the forward analysis [31]. XFEM has been proven adept in the modeling of multiple shaped inclusions/void and cracks with XFEM [32, 33], as well as in the modeling of arbitrarily-shaped objects as demonstrated by Benowitz and Waisman [34], and Jung and Taciroglu [35].

Complimentary to the forward problem, an appropriate optimization procedure need be enforced. Heuristic optimization [36] is particularly suited to such an end, since it allows for flexibility in the formulation of the forward problem, which need not be linear, convex, or smooth. Due to this feature, different forms of heuristic procedures have been adopted in the context of Structural Health Monitoring. Hunaidi [37] employs evolution-based Genetic Algorithms (GAs) for non-destructive assessment of pavements on the basis of surface waves tests; Farley et al. [38] adopt an artificial neural network approach for defect detection via ultrasonic signals; Lee et al. [39] formulate an inverse scattering problem on the basis of Particle Swarm Optimization; while Bernieri et al. [40] reconstruct cracks via eddy current testing and a machine learning approach.

For the solution of the inverse problem in the particular domain of flaw/crack detection, Rabinovich et al. [41, 42], combine and XFEM approach with GAs for crack identification in static and dynamic 2D problems. Waisman et al. [43] and Chatzi et al. [44], extend and experimentally validate the XFEM–GA scheme for identification of generalized flaw types. Sun at al. [45] presented an adaptive algorithm, once again relying on XFEM, able to detect multiple flaws without prior knowledge on their number by means of an Enhanced Artificial Bee Colony (EABC) algorithm [46] and a sweeping window method for dynamic problems [47]. Yan et al. [48] introduce a guided bayesian inference approach for detection of multiple flaws. Jung and Taciroglu employ XFEM for identification of an arbitrarily shaped scatterer embedded in elastic heterogeneous media [35]. Nanthakumar et al. [49] combine XFEM to the Multilevel Coordinate Search (MCS) method to detect cracks and voids in piezoelectric materials, while in a later work [50] they employ derivatives of the level sets for the optimization step in order to increase the robustness and efficiency of their method. In a more recent work [51], the same authoring team applies the XFEM–MCS scheme to the detection of multiple cracks in piezoelectric structures under dynamic electric loads. In Ma et al. [52] XFEM is incorporated in a three step algorithm for the detection of multiple flaw clusters. Finally, XFEM is employed in damage detection schemes for dams in the works of Alalade et al. [53] and Pirboudaghi et al. [54].

A characteristic feature of the aforementioned works is their confinement and demonstration in the two-dimensional domain. The extension in the third dimension comes with a number of challenges, some of which have recently been tackled in a robust 3D XFEM scheme introduced by the authoring team [55, 56]. This XFEM scheme was coupled with a Covariance Matrix Adaptation Evolution Strategy (CMA-ES) [57] in a first attempt to apply XFEM based crack detection to 3D problems [58]. In the present work, the proposed XFEM variant is combined to a multiscale optimization strategy consisting of a discontinuous step utilizing genetic algorithms and a continuous step utilizing the CMA-ES algorithm [57] in order to detect multiple cracks in 3D solids.

## Inverse problem formulation

Inverse problems aim at identifying the latent and unknown parameters of a system given measured information on its response and commonly, albeit not necessarily, a computational model of the system. The estimation of structural response for a prescribed set of model parameters using an available model structure may be considered as the forward problem. In the present case, this forward problem is solved via a 3D XFEM approach.

For the specific case of detection of multiple flaws in the form of cracks, the unknown parameter set comprises the number, location, shape, size and orientation of existing cracks in a structure. While various sensors may be employed for monitoring structural response, we here assume availability of strain information at specific locations along the structure obtained via conventional strain gauges. Due to its low cost and ease of deployment this monitoring option is often adopted, albeit distributed sensing alternatives, such as fiber optics solutions [59], may also be adopted.

The inverse formulation may then be summarized as the following optimization problem [41, 43]:

1 $$\begin{aligned}&\textit{Find } \theta _i \textit{ such that} \nonumber \\&\quad \mathcal {F} \left( \theta _i \right) \rightarrow \text {min} \end{aligned}$$

where $\theta _i$ is a set of parameters used to describe the number, location, shape, size and orientation of the cracks and $\mathcal {F}$ is the objective function given by:

2 $$\begin{aligned} \mathcal {F} \left( \theta _i \right) = \dfrac{\left\| \bar{{\varvec{\epsilon }}}^h \left( \theta _i\right) -\bar{{\varvec{\epsilon }}}^m \right\| }{\left\| \bar{{\varvec{\epsilon }}}^m \right\| } \end{aligned}$$

where $\bar{{\varvec{\epsilon }}}^h\left( \theta _i\right)$ are the numerically computed strains at the sensor locations and $\bar{{\varvec{\epsilon }}}^m$ are the measured strains at the same locations. The strain components for all sensor locations considered are arranged in vectors containing $n_c \times n_s$ elements, where $n_c$ is the number of components of the strain tensor ($n_c=9$ for the 3D case), and $n_s$ pertains to the number of sensors.

## Solution of the forward problem using XFEM

For the solution of the optimization problem posed in the previous section, several evaluations of the fitness function, for different values of the design variables, are required. These evaluations correspond to solutions of the forward problem, for different crack numbers, shapes, sizes and locations, and should be obtained in a robust and efficient way, ensuring minimization of the associated computational toll. In the present work, the extended finite element method (XFEM) [30], and in particular the variant introduced in Reference [56], is employed for the solution of forward problems. The method has already been successfully used in 2D crack detection schemes [41, 43] due to its ability to represent discontinuities without requiring any modification of the finite element mesh, a feature which is crucial for this category of applications where the forward problem has to be solved for a very large number of different crack configurations. In the following subsections, the forward problem is mathematically formulated and the solution method is presented.

**Fig. 1:**
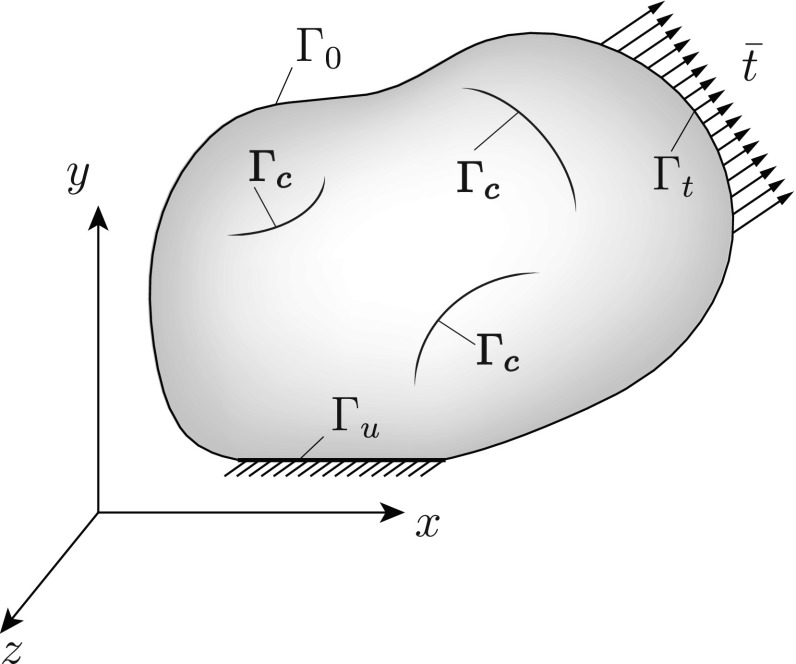
Cracked body and boundary conditions

### Problem statement

The problem consists of a linear elastic solid $\varOmega$ (Fig. 1) cracked at several locations and bounded by the boundary $\varGamma$ where:

3 $$\begin{aligned} \varGamma =\varGamma _0 \cup \varGamma _u \cup \varGamma _t \cup \varGamma _c \end{aligned}$$

$\varGamma _0$ is the part of the boundary where no boundary conditions are applied. $\varGamma _u$ is the part of the boundary where displacements $\bar{u}$ are imposed as Dirichlet boundary conditions. $\varGamma _t$ is the part of the boundary where surface tractions $\bar{t}$ are applied as Neumann conditions. $\varGamma _c$ is the surface of the cracks.

The equilibrium equations are formulated in weak form as:

Find a kinematically admissible displacement field ${\varvec{u}}\in \mathcal {U}$ such that $\forall {\varvec{v}}\in \mathcal {V}$

4 $$\begin{aligned} \int _{\varOmega } {\varvec{\epsilon }}({\varvec{u}}): {\varvec{D}}: {\varvec{\epsilon }}({\varvec{v}}) \ d\varOmega = \int _{\varOmega } {\varvec{b}}\cdot {\varvec{v}}\ d\varOmega + \int _{\varGamma _t} \bar{{\varvec{t}}}\cdot {\varvec{v}}\ d\varGamma \end{aligned}$$

where:

5 $$\begin{aligned} \mathcal {U} = \left\{ {\varvec{u}}|{\varvec{u}}\in \left( H^1\left( \varOmega \right) \right) ^3,{\varvec{u}}= \bar{{\varvec{u}}} \text { on } \varGamma _u \right\} \end{aligned}$$

and

6 $$\begin{aligned} \mathcal {V} = \left\{ {\varvec{v}}|{\varvec{v}}\in \left( H^1\left( \varOmega \right) \right) ^3,{\varvec{v}}= \mathbf {0} \text { on } \varGamma _u \right\} \end{aligned}$$

Functions of $H^1\left( \varOmega \right)$ are implicitly discontinuous along the crack surfaces.

In the above, ${\varvec{\epsilon }}$ is the small strain field, ${\varvec{D}}$ is the elasticity tensor and ${\varvec{b}}$ is the applied body force per unit volume.

### Crack representation

As is commonly the case in XFEM [60–62], cracks are represented implicitly using the level set method. Level set functions, denoted as $\phi$ and $\psi$, are defined for an arbitrary point $\mathbf {x}$ as follows:

- $\phi$ is the signed distance from the crack surface defined as:
  7 $$\begin{aligned} \phi \left( \mathbf {x}\right) = \min _{\bar{\mathbf {x}}\in \varGamma _c}{\left\| \mathbf {x}-\bar{\mathbf {x}}\right\| \text {sign}\left( \mathbf {n}^+\cdot \left( \mathbf {x}-\bar{\mathbf {x}}\right) \right) } \end{aligned}$$
  where $\mathbf {n}^+$ is the outward normal to the crack surface and $\text {sign}\left( \right)$ is the $\text {sign}$ function.
- $\psi$ is a signed distance function such that $\nabla \phi \cdot \nabla \psi =0$ and $\phi \left( \mathbf {x}\right) =0$ and $\psi \left( \mathbf {x}\right) =0$ defines the crack front.

Additionally, a polar coordinate system is defined along the crack front with coordinates [60–62]:

8 $$\begin{aligned} r=\sqrt{\phi ^2+\psi ^2}, \quad \theta =\arctan \left( \frac{\phi }{\psi }\right) \end{aligned}$$

These coordinates refer to a plane normal to the crack front.

### Discretization

The weak form is discretized using a stable XFEM variant introduced in the authors’ previous works [55, 56]. The method was shown to provide increased accuracy and improved conditioning when compared to standard XFEM. Moreover, it enables the use of higher order enrichment functions in 3D linear elastic fracture mechanics.

Perhaps the most critical feature of extended and generalized finite element methods (X/GFEM) is the enrichment of the FE approximation with functions which are able to represent known features of the solution. Enrichment is realized by employing the partition of unity (PU) method [63]:

9 $$\begin{aligned} \mathbf {u}\left( \mathbf {x}\right) =\underbrace{\sum \nolimits _{\forall I} N_I\left( \mathbf {x}\right) \mathbf {u}_I}_{\text {FE approximation}} +\underbrace{\sum \nolimits _{\forall I} N_I^*\left( \mathbf {x}\right) \varPsi \left( \mathbf {x}\right) \mathbf {b}_I}_{\text {enriched part}} \end{aligned}$$

where $N_I\left( \mathbf {x}\right)$ are the FE interpolation functions, $\mathbf {u}_I$ are FE degrees of freedom (dofs), $N_I^*\left( \mathbf {x}\right)$ is a basis of functions that form a partition of unity, $\varPsi \left( \mathbf {x}\right)$ are the enrichment functions and $\mathbf {b}_I$ are the enriched degrees of freedom.

While in PU-FEM enrichment is applied globally to all the FE nodes, in XFEM enrichment is only applied locally to approximate local phenomena such as cracks and discontinuities. This can be achieved by appropriately defining the set of enriched nodes, as will be done in the following.

In linear elastic fracture mechanics two different enrichment functions are employed, the modified Heaviside or jump enrichment functions:

10 $$\begin{aligned} H(\phi )=\left\{ \begin{array}{ll} 1&{}\quad \text {for}\quad \phi \ge 0 \\ -1&{}\quad \text {for}\quad \phi < 0 \end{array}\right. \end{aligned}$$

which are used to represent the displacement jump along the crack surfaces, and the asymptotic or tip enrichment functions:

11 $$\begin{aligned} F_j\left( r,\theta \right)= & {} \left\{ \sqrt{r} \sin \frac{\theta }{2}, \sqrt{r} \cos \frac{\theta }{2}, \sqrt{r} \sin \frac{\theta }{2} \sin \theta , \right. \nonumber \\&\left. \sqrt{r} \cos \frac{\theta }{2} \sin \theta \right\} \end{aligned}$$

which are used to represent the asymptotic fields around the crack front.

Since enrichment is applied locally, the nodal sets where each enrichment function is used have to be appropriately selected:

- Jump enrichment is used for nodes belonging to elements that are divided in two parts by the crack surface.
- Tip enrichment is used for nodes belonging to elements that contain the crack front (topological enrichment), or for nodes that lie in a certain distance (enrichment radius) from the crack front (geometrical enrichment). In the first case sub optimal convergence rates are obtained [64, 65]. In the second, while optimal convergence is achieved, conditioning problems are caused, for the solution of which, special techniques are necessary [65–67].

**Fig. 2:**
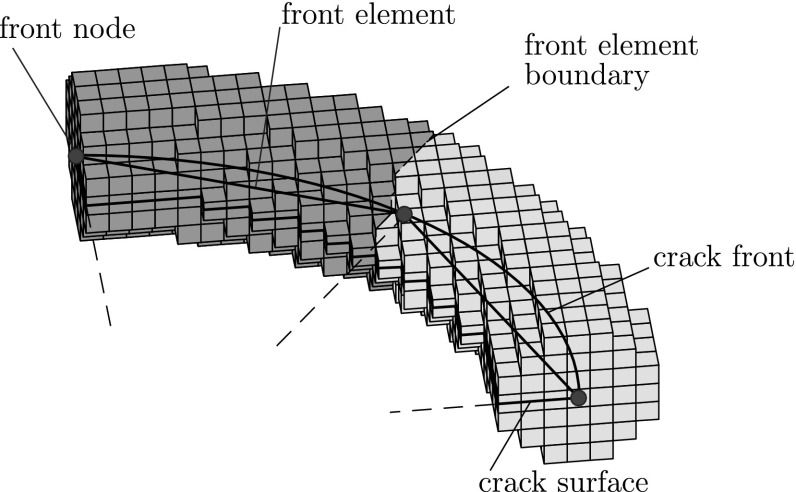
Front elements, nodes and boundaries

Functions $N_I^*\left( \mathbf {x}\right)$ used for the partition of unity enrichment are typically selected to coincide with the FE shape functions $\left( N_I\left( \mathbf {x}\right) \equiv N_I^*\left( \mathbf {x}\right) \right)$. In the variant used herein however, an alternative definition is used which has been shown [55, 56] to provide improved conditioning of the resulting stiffness matrices. More specifically, a superimposed mesh of special elements discretizing the crack front is introduced, as illustrated in Fig. 2 and the shape functions corresponding to those front elements are used as a basis for the PU enrichment.

**Fig. 3:**
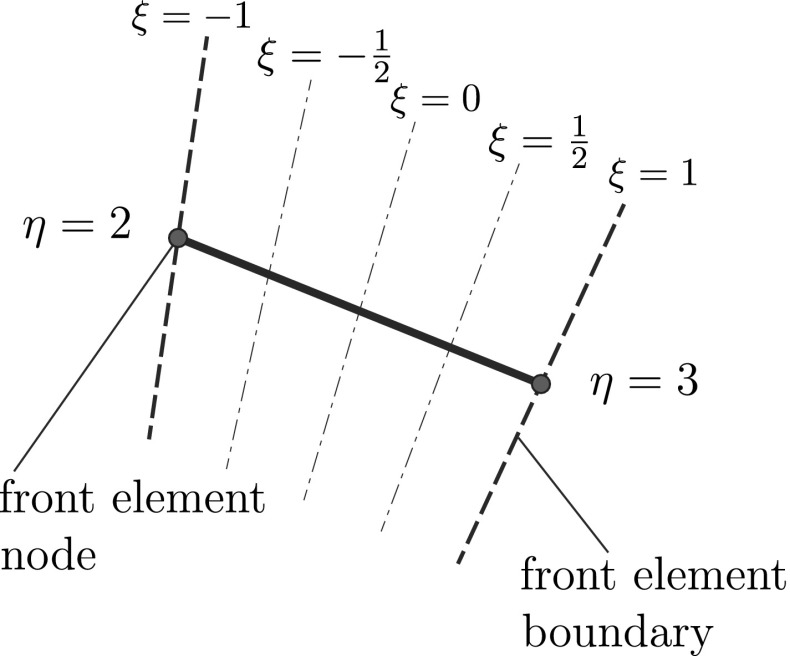
Local coordinate $\xi$ of the front elements

The shape functions of the front elements are defined as simple 1D FE shape functions:

12 $$\begin{aligned} \mathbf {N}^g\left( \xi \right) =\left[ \frac{1-\xi }{2}\quad \frac{1+\xi }{2}\right] \end{aligned}$$

where $\xi$ is the local coordinate of the superimposed element (Fig. 3). This parameter is defined in detail in References [55, 56].

Blending problems between the standard and the enriched part of the approximation [68–70] are dealt with using the techniques developed in the works of Fries [68] and Ventura et al. [69] and applied in a 3D framework in our previous works [55, 56]. These introduce a weight function $\varphi \left( \mathbf {x}\right)$ that assumes a value of unity for the fully enriched elements, and linearly fades to zero for the blending elements. The blending area, along which the weight function fades to zero, can consist of one or several layers of elements [56].

The displacement approximation for the method is:

13 $$\begin{aligned} \mathbf {u}\left( \mathbf {x}\right)&=\sum _{I\in \mathcal {N}} N_I\left( \mathbf {x}\right) \mathbf {u}_I \nonumber \\&\quad + \bar{\varphi }\left( \mathbf {x}\right) \sum _{J\in \mathcal {N}^j} N_J\left( \mathbf {x}\right) (H\left( \mathbf {x}\right) - H_J) \mathbf {b}_J \nonumber \\&\quad + \varphi \left( \mathbf {x}\right) \left( \sum _{K\in \mathcal {N}^s} N_K^g\left( \mathbf {x}\right) \sum _{j} F_{j}\left( \mathbf {x}\right) \right. \nonumber \\&\quad \left. - \sum _{T\in \mathcal {N}^t} N_T\left( \mathbf {x}\right) \sum _{K\in \mathcal {N}^s} N_K^g\left( \mathbf {x}_T\right) \sum _{j} F_{j}\left( \mathbf {x}_T\right) \right) \mathbf {c}_{Kj} \end{aligned}$$

where $\mathcal {N}$ is the set of all nodes in the FE mesh. $\mathcal {N}^j$ is the set of jump enriched nodes. This nodal set includes all nodes whose support is split in two by the crack and in addition belong to elements where the weight function $\bar{\varphi }\left( \mathbf {x}\right)$ assumes values greater than zero. $\mathcal {N}^t$ is the set of tip enriched nodes. This nodal set includes all nodes that belong to an element with at least one node inside the enrichment radius. $\mathcal {N}^s$ is the set of nodes in the superimposed mesh.

## Parametrization and constraints

Since the present work is only one of the first attempts to extend flaw detection schemes [41, 43, 44] in 3D, some simplifications are made in order to reduce the complexity of the general problem. Two main simplifications are made, with regard to the crack geometries and interactions.

The first aims at reducing the number of parameters used to represent crack geometries by only employing elliptical cracks for the forward problem, and approximating cracks of different shapes by appropriately varying the ellipse parameters. Although this approach may seem somehow limited, it provides the possibility to model a variety of crack shapes, while requiring a relatively small number of parameters to describe each crack.

A second simplification is assumed with respect to the interactions between different cracks. Although multiple cracks are considered, we herein only investigate cases where the minimum distances between the different cracks are larger than some predefined value. The above approach is necessary in order to avoid crack intersections which would pose problems in the solution of the forward problem with XFEM, since the treatment of intersecting cracks in 3D can be problematic.

The above simplifications can be overviewed as follows. The scheme developed in this work aims at determining the number and locations of existing cracks and roughly estimating their sizes and shapes. The accurate determination of the geometrical shapes of the cracks, including crack intersections, exceeds the above aim and in addition would be limited by the amount and accuracy of the available structural response measurements.

### Parametrization

**Fig. 4:**
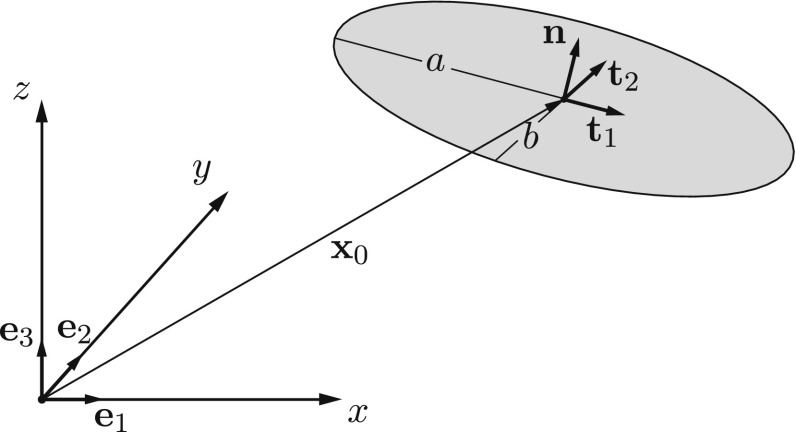
Parametrization of an elliptical crack

The parameters involved in the definition of each elliptical crack are the coordinates of its center point $\mathbf {x}_0$ ($\left\{ x_0,y_0,z_0\right\}$), the angles of rotation about the three axes $\phi _x, \phi _y$ and $\phi _z$ and lengths *a* and *b*. Angles $\phi _x, \phi _y$ and $\phi _z$ are used to produce vectors $\mathbf {n}$, $\mathbf {t}_1$ and $\mathbf {t}_2$ by rotating unit vectors $\mathbf {e}_1, \mathbf {e}_2$ and $\mathbf {e}_3$. All of the above parameters are illustrated in Fig. 4.

### Constraints for a single crack

While the range of values assumed by the design variables may be restricted by upper and lower bounds, for complex structure geometries invalid crack locations may still be generated which would result in unnecessary solutions of the forward problem. In order to avoid such occurrences, a method of determining the relative position of the cracks with respect to the structure is introduced herein. This method represents the boundaries of the structure via implicit functions and evaluates this function for several points on the crack surface.

#### Radial basis functions

The implicit functions used in the present work are radial basis functions [71] (RBF) and they are defined so as to assume negative values in the interior of the structure, positive values in the exterior and a value equal to zero on the structure boundaries.

Radial basis functions are constructed from a set of points $\mathbf {x}_i, i=1, \ldots , N$ lying on and off the surface to be described. In general they assume the form:

14 $$\begin{aligned} s\left( \mathbf {x}\right) =p\left( \mathbf {x}\right) + \sum _{i=1}^N \lambda _i R\left( \left\| \mathbf {x}-\mathbf {x}_i\right\| \right) \end{aligned}$$

where *p* is a low degree polynomial: $p\left( \mathbf {x}\right) =\left\{ a_1, a_2, \ldots , a_l\right\} \cdot \left\{ p_1, p_2, \ldots , p_l\right\} ^{T}$ where $a_i$ are coefficients to be determined, $p_i$ are the elements of the polynomial basis and *l* is the number of polynomial terms used *R* is the basic function, common choices for this function are:

- The thin plate spline: $R\left( r\right) =r^2 \log \left( r\right)$
- The Gaussian: $$R\left( r\right) = e^{-c r^2}$$
- The multiquadric: $$R\left( r\right) = \sqrt{r^2+c^2}$$
- The biharmonic spline: $R\left( r\right) = r$
- The triharmonic spline: $R\left( r\right) = r^3$

in the above $r=\left\| \mathbf {x}-\mathbf {x}_i\right\|$. The variable *r* in this case is not to be confused with the polar coordinate used in Sect. 3.

$$\lambda _i$$
are coefficients to be determined.

By employing the known values of the function $s_i$ at points $\mathbf {x}_i$ a system of equation can be created:

15 $$\begin{aligned} \left( \begin{array}{cc} \mathbf {A} &{} \mathbf {P} \\ \mathbf {P}^{T} &{} \mathbf {0} \end{array}\right) \left( \begin{array}{c} {\varvec{\lambda }} \\ \mathbf {a} \end{array}\right) = \left( \begin{array}{c} \mathbf {s} \\ \mathbf {0} \end{array}\right) \end{aligned}$$

where

$$\begin{aligned} \begin{array}{ll} A_{ij}=R\left( \left\| \mathbf {x}_i-\mathbf {x}_j\right\| \right) ,&{} i,j=1, \ldots , N \\ P_{ij}=p_j\left( \mathbf {x}_i\right) ,&{} i=1, \ldots , N, j=1, \ldots , l \end{array} \end{aligned}$$

The solution of the above system yields the values of the coefficients $a_i$ and $\lambda _i$ which in term make possible the evaluation of the RBF at any given point.

#### Determination of invalid cracks

**Fig. 5:**
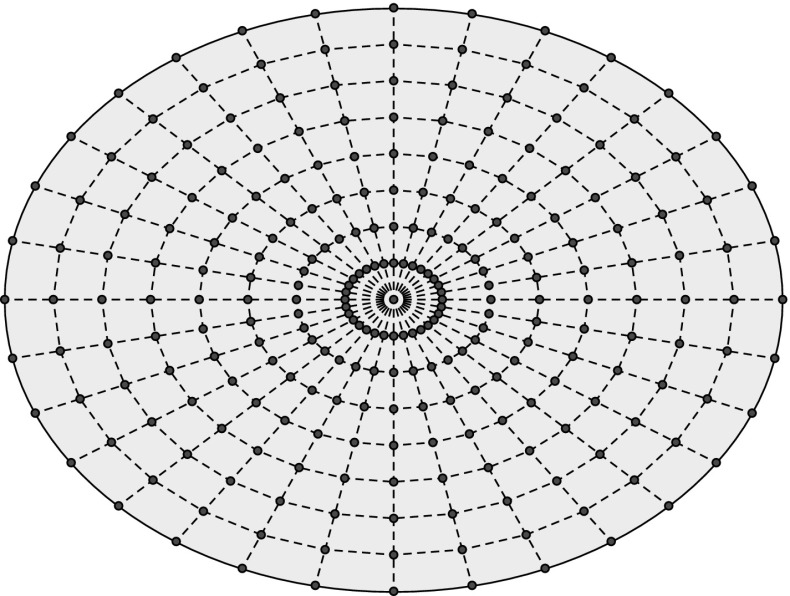
Control points on the crack surface

Once the RBF representation of the structure has been constructed, a set of control points lying on the crack surface is generated for each candidate crack. For the elliptical cracks considered in the present work, those points are generated according to the pattern illustrated in Fig. 5. The relative position of the crack with respect to the structure can be determined from the signs of the RBF at the control points. For instance, if the sign of the RBF is negative for all the control points then crack lies entirely inside the structure.

Moreover, from the signs and values of the RBF at the control points some other cases can be identified:

- When only a fraction of the control points assume positive values, then the corresponding crack intersects the structure boundary and, since such cases are also of interest, it is considered valid. However, the percentage of positive values should lie within certain bounds in order to avoid situations where a very small or a very large part of the crack lies outside the structure. Those cracks, besides being physically meaningless, could potentially cause numerical problems and should be discarded.
- If the majority of points assume low values (below a predefined tolerance), the crack is considered invalid since an actual crack would not lie on the structural boundary. This case could also cause numerical problems.
- If the RBF repeatedly alternates in sign along a line of points (Fig. 5), then the crack is discarded since only simple intersections of the crack with the boundary are considered.

Considering the above, some further remarks can be made regarding the definition and use of the RBF in the present application:

- Since the RBF values of several points are taken into account in order to determine the position of the crack with respect to the structure, the zero iso-surface of the function does not need to coincide very accurately with the structure boundaries. As a result the number of points needed to define the RBF can be kept relatively small, making the generation and evaluation of the function faster.
- The RBF function can be modified in order to restrict the search space in a part of the structure where the cracks are expected to be lying, thus making the whole procedure faster.

### Constraints for multiple cracks

The procedure described above for a single crack has to be applied for each individual crack in the case of multiple cracks. Additionally, overlapping or intersecting cracks have to be detected and discarded.

#### Detection of overlapping cracks

**Fig. 6:**
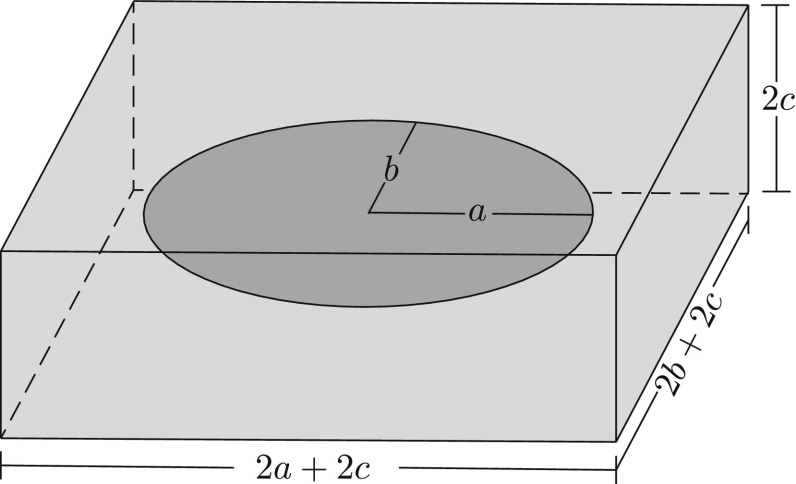
Bounding box used to prevent crack intersections

In order to detect overlapping or intersecting cracks a bounding box is first defined for each crack as in Fig. 6. The sides of the bounding boxes are given the values $2a_i+2c$, $2b_i+2c$ and 2*c* where *a* and *b* are the lengths defining the corresponding crack. Parameter *c* should be attributed a large enough value in order to ensure that enriched elements belonging to different cracks do not overlap. Subsequently, the separating axis theorem [72] is employed to determine whether two bounding boxes intersect.

For a given set of candidate cracks the detection of intersections is achieved by investigating all possible crack pairs and determining weather the corresponding bounding boxes intersect. If two bounding boxes are found to intersect, then one of the corresponding cracks is discarded. In our current implementation the selection of the crack to be discarded is being done arbitrarily since it is assumed the cracks will be either overlapping or in close proximity therefore either of the cracks will eventually converge to the actual crack if retained.

A more refined method for performing the above selection would consist of evaluating the fitness function for both cracks and eliminating the crack leading to the worst value. Such a criterion might lead in faster convergence of the optimization process in the expense of increasing the numerical cost of the evaluation of individual crack configurations. Nevertheless, a detailed comparison would exceed the purposes of the present work.

## Inverse problem solution

For the solution of the inverse problem a multiscale strategy, similar to Reference [46] is employed, which utilizes two different optimization algorithms. In what follows, the two algorithms are first briefly described with the proposed hybrid strategy introduced next.

### Genetic algorithms

Genetic algorithms (GAs) are a category of optimization tools inspired by biological evolution [73, 74]. Solutions to optimization problems are obtained by iteratively improving a set of candidate solutions in an attempt to mimic natural evolution processes. Following the GA terminology, the set of candidate solutions is termed the population of individuals, while each iteration corresponds to a generation. Each individual in the population is represented by set of genes whose numerical equivalent is a binary array. Moreover, in order to mimic natural selection through survival of the fittest, a fitness value is assigned to each individual by evaluating the fitness function which usually coincides with the objective function [75]. Typically the following steps take place in a genetic algorithm:

Initialization: Once the number of parameters and the population size have been set the initial population is generated, usually randomly.
Selection: The fitness function is evaluated for all the individuals in each generation and only a percentage of the population, corresponding to the highest fitness values, is selected to form the next generation.
Reproduction: During this step the fittest individuals from each generation reproduce to form the next generation, two processes are involved in this reproduction:

Crossover: The genes of two individuals (parents) are combined, through recombination of the bits corresponding to their bit representation, to form an offspring.
Mutation: During the reproduction procedure, some bits are randomly flipped in order to simulate mutations that occur in the biological reproduction process.

During this step another practice, called elitism, is commonly used which consists of allowing the fittest individual or individuals to survive, unaltered, in the next generation.

The above steps are repeated until some prescribed termination criteria are met. The parameters involved in the above steps are user-defined and include the population size, crossover rate, mutation rate and the termination criteria. The most widely adopted termination criteria include the definition of a maximal number of generations, a predefined target value for the fitness function, as well as a maximal consecutive number of generations without improvement in the fitness values.

### Covariance matrix adaptation evolution strategy (CMA-ES)

In this method [57, 76] candidate solutions are generated from a multivariate normal distribution whose parameters, namely the distribution mean, covariance matrix and step size, are updated such that the probability of obtaining improved solutions is increased.

Distribution mean: The distribution mean is updated so that the probability of successful candidate solutions is increased. This is achieved by setting the mean in each iteration equal to the weighted average of a predefined number of candidates with the best fitness values from the previous iteration.
Covariance matrix: The covariance matrix is updated so that the probability of successful search directions is increased and in addition information from previous generations is utilized.
Step size: The step size is adjusted in order to avoid premature convergence while yet ensuring that the algorithm converges fast enough.

### The proposed multiscale strategy

The basic idea behind the strategy proposed herein, is similar to the one introduced in Sun et al. [46]. In particular, a two step procedure is adopted where in the first step a discrete optimization algorithm is used to obtain the number and approximate location, size, and orientation of the cracks while in the second step a continuous optimization algorithm is employed to refine the values of the parameters obtained in the first step. The discrete optimization step is employed in order to reduce the complexity of the original problem and obtain an approximate solution which is used as an initial guess for the continuous step where a more accurate solution can be obtained. The two steps are described in detail in the following.

#### Discrete optimization step

In the first step of the procedure, in which Genetic Algorithms are used as an optimization tool, the number of cracks is identified, therefore topological variables [45] are employed to activate/deactivate candidate cracks. Moreover, the original identification problem is simplified in order to minimize the number of parameters to be identified thus accelerating the convergence to the approximate solution. The reduction of the number of parameters is achieved in two ways, firstly by assuming the shape of the cracks to be detected circular rather than elliptical and secondly by reducing the number of binary digits used to represent each of the parameters involved in the optimization process.

At this stage, the parameters described in Sect. 4.1 are encoded as follows:

$$x_0,y_0,z_0$$
The coordinates of the center of the ellipses are encoded as:
  16 $$\begin{aligned} p_i=p_{{ imin}}+\left( p_{{ imax}}-p_{{ imin}}\right) \theta _i \end{aligned}$$
  where $p_i$ are the coordinates, $p_{{ imin}}$ and $p_{{ imax}}$ are the minimum and maximum values allowed for these coordinates and $\theta _i$ are the design variables used in the genetic algorithm. In order to represent variables $\theta _i$, *n* binary digits are used and the resulting values are divided by $2^n$ so that the variables assume *n* possible values between 0 and 1. The number of digits defines the total number of possible crack locations and should be chosen according to the geometry of the solid. It should be noted that a different number of digits for each variable can be used. The minimum and maximum values define a box which should contain the whole domain of interest.
*a*, *b*: In the first step of the procedure cracks are considered to be circular, therefore the two ellipse parameters are equal and a single variable is required for their representation. The encoding used for this variable is the same as the one used for the previous variables (Eq. 16) and the minimum and maximum values should be chosen according to the expected size of the cracks to be detected.
$$\phi _x, \phi _y, \phi _z$$
Since cracks are assumed to be circular only the first two angles are used at this stage. The encoding used is again that of Eq. (16) and the minimum and maximum values are set to 0 and $\pi$ respectively while the number of digits used is $n=2$ which results in 4 possible values for each angle. For these parameters variables are divided by $2^n+1$ so that the value $\pi$ is not included in the possible values for the angles since it is equivalent to the value 0. For the specific choice $n=2$ the possible values for each angle are 0, $\pi /4$, $\pi /2$ and $3 \pi /4$.

At this step of the algorithm, candidate solutions that violate the constraints described in Sect. 4 are penalized by being assigned large fitness values. For those solutions the forward problem does not have to be solved. Also, it is possible that the solution produced by this step contains two or more overlapping cracks. Although those cracks might be activated through their corresponding topological variables, the procedure described in Sect. 4.3 will discard all but one of the overlapping cracks and therefore the value of the fitness function obtained will correspond to a single crack at that specific location. At the end of the step cracks that have been discarded through the above process are considered inactive and as a result are not considered in the next step of the optimization procedure.

#### Continuous optimization step

In the second part of the multiscale strategy, the results obtained in the previous stage are used as an initial solution for the CMA-ES algorithm. The number of cracks is assumed to have been correctly determined in the previous step. Furthermore, the scaling of the parameters and the initial step size used in the algorithm are chosen so that the search space is confined in a small part of the original search space around the initial values. This is achieved by using the following encoding for the parameters of the problem.

17 $$\begin{aligned} p_i=p_{i0} + \dfrac{\theta _i}{10} dp_i \end{aligned}$$

where $p_{i0}$ are the initial values of the parameters obtained in the previous step, $dp_i$ are the half lengths of the search space (in the direction of each parameter) and $\theta _i$ are the design variables used in the algorithm.

In the above, half lengths of the search spaces are given a value equal to the distance of two consecutive possible values (length of the search space for each variable divided by $2^n$) of the previous step of the algorithm. The design variables are initialized to zero and the step size is set to $\sigma = 3$ which implies that the final solution lies in the interval $0 \pm 2 \sigma = 0 \pm 6$.

For the parameters that were omitted in the first step (*a* and $\theta _z$) a slightly different approach is used. Regarding the parameters of the ellipse, the value computed in the first step (were the two parameters were assumed equal) is used as an initial value for parameter *b* for which the encoding of Eq. (17) is used. Parameter *a* which should be larger than or equal to parameter *b* is obtained as the sum of parameter *b* and an additional parameter $a_{inc}$:

18 $$\begin{aligned} a=b + a_{{ inc}} \end{aligned}$$

where the additional parameter is computed as:

19 $$\begin{aligned} a_{{ inc}} = \dfrac{\left| \theta _a\right| }{10} d_a \end{aligned}$$

In the above, $\theta _a$ is the design variable corresponding to $a_{{ inc}}$, $d_a$ is the maximum allowed difference between *a* and *b* and the absolute value is used to prevent $a_{{ inc}}$ from assuming negative values and therefore *b* from assuming larger values than *a*.

Regarding angle $\phi _z$, the following encoding is employed:

20 $$\begin{aligned} \phi _z = \dfrac{\theta _{\phi _z}}{10} \dfrac{\pi }{2} \end{aligned}$$

in order to restrict the possible values of the angle in the interval $\left[ -\pi / 2, \pi /2 \right]$.

At this step candidate solutions that violate constraints are re-sampled.

#### Discussion

In the strategy described above, the problem to be solved in each individual step is of reduced complexity in comparison to the original problem definition. In the first step, the dimension of the search space is significantly reduced by removing some of the problem parameters, and reducing the number of binary digits used to represent the remaining ones. In the second step, the search space is restricted in a small region around the solution obtained in the previous step. Moreover, in the second part of the algorithm the number of cracks is considered to have already been determined, and as a result the problem is further simplified. Without these simplifications, the complexity of the problem would render convergence extremely slow, or even impossible.

## Numerical examples

The potential of the proposed method is demonstrated in three numerical examples involving the detection of multiple cracks in solids of varying geometrical complexity.

The forward problem is solved using topological enrichment in order to reduce the computational cost associated with the numerical integration of the asymptotic enrichment functions. The XFEM variant used for the solution of the forward problems is still advantageous in this case since as shown in Reference [56] it provides improved conditioning and accuracy compared to standard XFEM.

The additional meshes required to discretize the crack front are automatically generated by dividing the circumference of the candidate cracks in segments of equal length, the approximate length of those segments is set to 2*h*, where *h* is the mesh parameter. Since edge cracks are also considered it is possible that several of the elements created lie entirely outside the solid considered therefore resulting in zero stiffness matrix entries which of course do not affect the solution.

For the evaluation of constraints, as mentioned in Sect. 4.2, the structure boundaries are represented using radial basis functions based on biharmonic functions and linear polynomials. Moreover, parameter *c* used in the definition of the bounding boxes described in Sect. 4.3.1 is given the value 5*h*. This value may seem large compared to the one required for standard XFEM in 2D, however the following factors need to be taken into account:

- In the XFEM variant used, the set of tip enriched nodes is larger than in standard XFEM since it involves nodes belonging to elements which lie along the layer surrounding the elements containing the crack front. Although those additional nodes and elements do not result in additional dofs, they are considered enriched and therefore have to be associated to one of the cracks. In addition, for curved crack fronts and unstructured meshes, additional elements might be characterized as enriched due to some of their nodes being enriched. Therefore the distance was extended to avoid such occurrences and to ensure the presence of at least one standard element between two enriched elements.
- It is considered that cracks are far enough so that no interaction between cracks takes place and that cracks which are in close proximity can be approximated by a single larger crack. As a result, the allowed distance between cracks can be further increased to prevent evaluations of the forward problem for cases that are not of interest and to avoid the aforementioned numerical problems.

Due to the stochastic nature of the algorithms used, the problems were solved 10 times and in the following representative runs from each problem are presented.

The method used for the forward problem was implemented in a C++ code utilizing the Gmm++ library [77] for linear algebra operations. The unstructured meshes used were generated using the gmsh mesher [78] and results were visualized using Paraview [79, 80].

For the optimization algorithms the MATLAB ga function and the MATLAB implementation of the CMA-ES algorithm [57, 81] developed by the Koumoutsakos group (CSE Lab), at ETH Zurich were used.

### Detection of two edge cracks in a unit cube

**Fig. 7:**
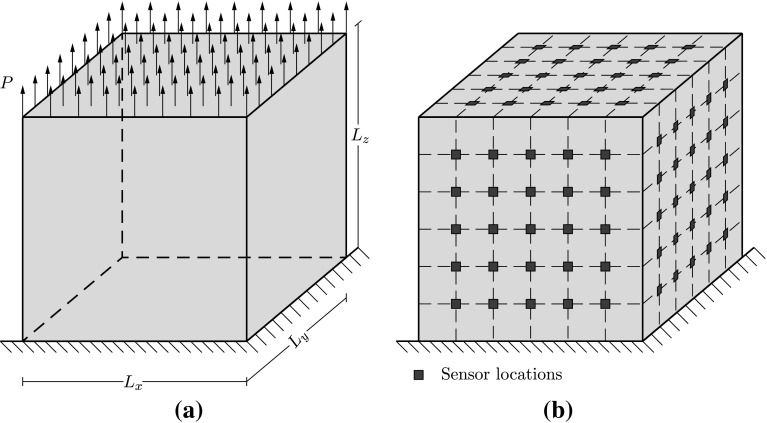
Unit cube. **a** Geometry and loading, **b** sensor locations. The geometry and load parameters are defined as $L_x=L_y=L_z=1$ unit and $P=1$ unit

The first example involves the detection of two edge cracks in a unit cube. The cube is fixed at one side and subjected to a uniform load at the other side as illustrated in Fig. 7a. The geometry parameters are defined as $L_x=L_y=L_z=1$ unit and the load has a unit value ($P=1$ unit). Academic material properties $E=200{,}000$ units and $\nu =0.2$ are used. The cube is meshed with a structured mesh consisting of $51 \times 51 \times 51$ tetrahedral elements. A network of $5 \times 5$ sensors is assumed to be located in each free face of the cube (Fig. 7b)). The strains measured by those sensors are simulated using a finer mesh of $101 \times 101 \times 101$ tetrahedral elements. The location of the cracks is shown in Fig. 10.

**Fig. 8:**
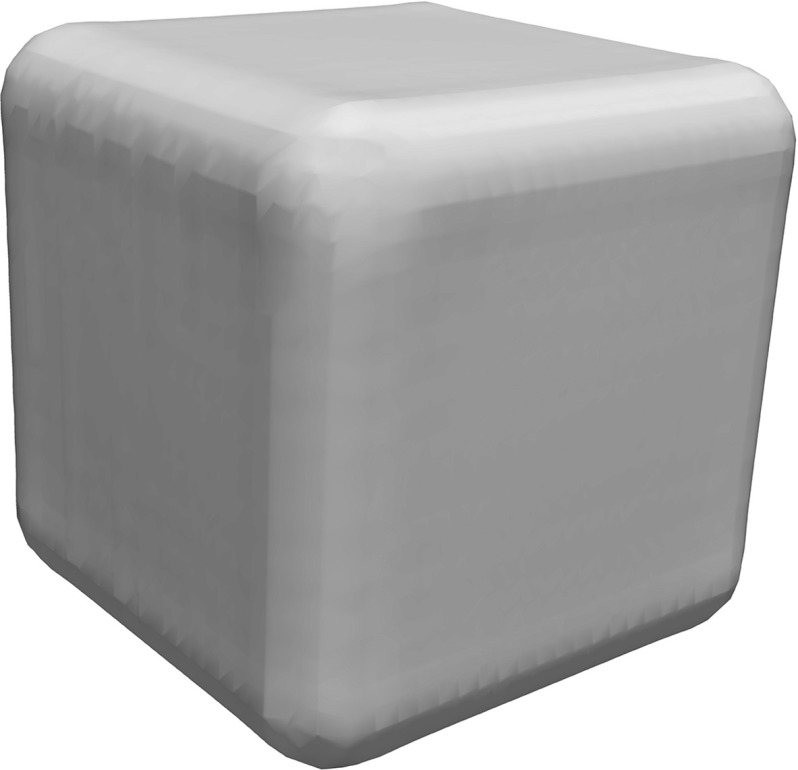
Unit cube, zero iso surface of the radial basis function used to describe the cube boundaries for the imposition of constraints

The RBF representation of the cube used for the evaluation of constraints in created using a set of $10 \times 10$ points on each edge of the cube. In Fig. 8 the zero iso surface of this RBF representation is illustrated. As can be seen the zero iso surface is not an accurate representation of the boundaries of the cube since it is only used to determine the relative location of candidate cracks with respect to the structure.

The limits for the parameters used in the first step of the optimization procedure of Sect. 5.3 were set to $x_{0\mathrm{min}}=0$ units, $x_{0\mathrm{max}}=1$ unit, $y_{0\mathrm{min}}=0$ units, $y_{0\mathrm{max}}=1$ unit, $z_{0\mathrm{min}}=0$ units, $z_{0\mathrm{max}}=1$ unit, $a_{\mathrm{min}}=b_{\mathrm{min}}= 0.15$ units and $a_{\mathrm{max}}=b_{\mathrm{max}}= 0.30$ units. Three binary digits (8 possible values) where used for the representation of parameters $x_0$, $y_0$, $z_0$ and two (4 possible values) for the rest of the parameters. The maximum number of cracks allowed in the medium is set to four and through the use of topological variables can be adjusted to the actual number of cracks (two). The population size was set to 40 individuals, the mutation rate was set to 0.4 in order to prevent the algorithm from converging to local minima and the optimization was set to run for 2000 evaluations of the fitness function.

For the second part of the procedure the default parameters of the CMA-ES algorithm are adopted resulting in a population of 12 individuals. The scaling of parameters defined in Subsection 5.3.2 results in each variable assuming values in the interval $\left[ -10,10\right]$ which would require an initial step size equal to $\sigma _0=6$. However, since the initial values of the parameters should already be close to the actual solution the initial step size is given a smaller value equal to $\sigma _0=3$. The maximum allowed difference between the two parameters of the ellipse is set to $d_a=0.10$ units. The CMA-ES algorithm is set to run for 2000 evaluations of the forward problem.

**Fig. 9:**
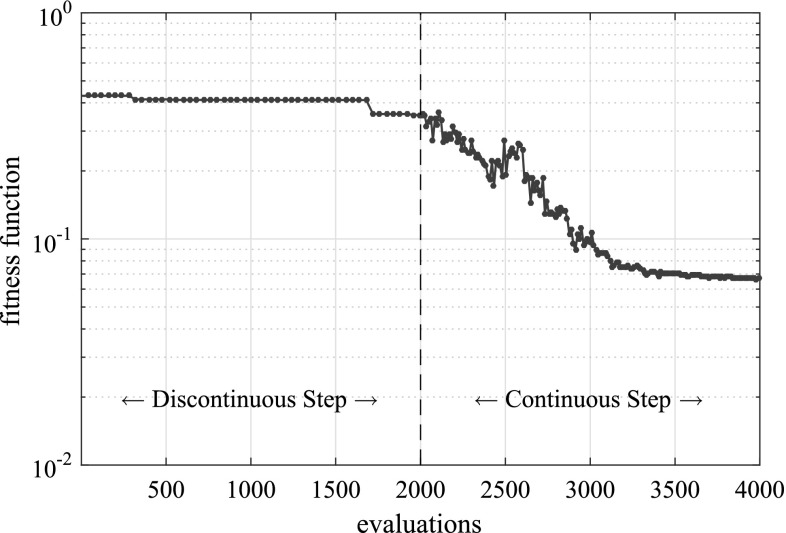
Convergence of the proposed multiscale strategy for the problem of a unit cube with multiple cracks

**Fig. 10:**
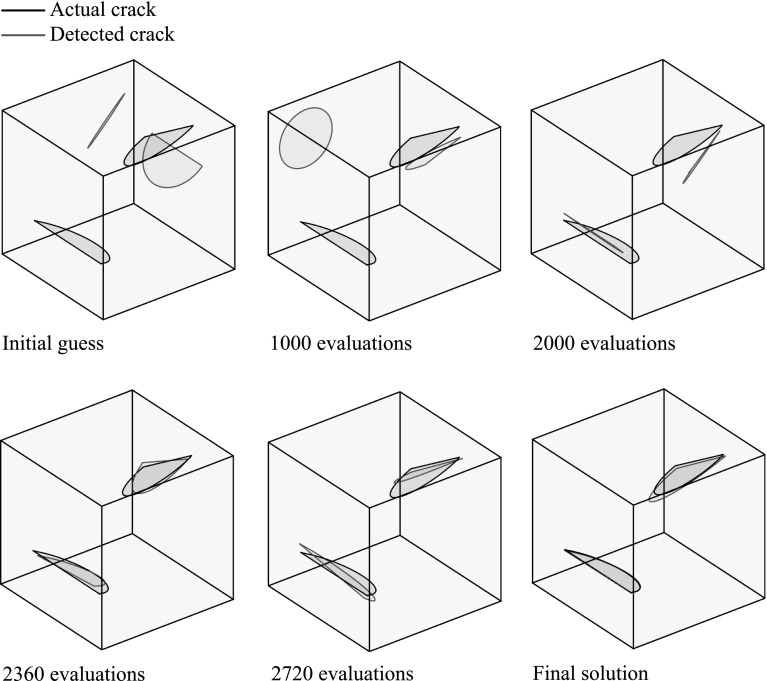
Best candidate solution after different numbers of evaluations for the problem of a unit cube with multiple cracks

In Fig. 9 the fitness function value achieved by the best individual of the population is given as a function of the number of evaluations of the fitness function, while in Fig. 10 the best solution after different numbers of evaluations is illustrated. In Table 1 the actual and detected values of the parameters describing the crack geometry are provided.

### Detection of three edge cracks in a beam under three point bending

**Fig. 11:**
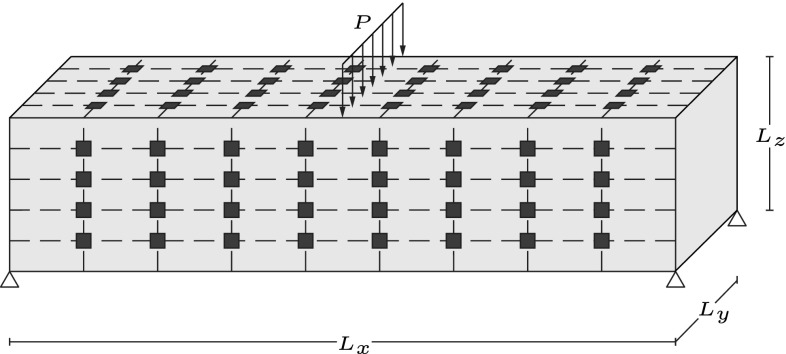
Geometry, loading and sensor locations for the beam under three point bending. The geometry and load parameters are defined as $L_x=0.6$ units, $L_y=0.15$ units, $L_z=0.15$ units and $P=1$ unit

In this example a beam under three point bending,as illustrated in Fig. 11, is considered with edge cracks in three different locations. The geometry parameters are defined as $L_x=0.6$ units, $L_y=0.15$ units, $L_z=0.15$ units and the load is given a unit value ($P=1$ unit). Academic material properties $E=200,000$ units and $\nu =0.3$ are used. A network of $4 \times 8$ sensors is assumed to be located in each of the long sides of the beam (Fig. 11).

**Table 1:** Actual and detected values for the parameters describing crack geometries for the problem of a unit cube with multiple cracks

Parameter	Actual value		Identified value	
	Crack 1	Crack 2	Crack 1	Crack 2
$x_0$	0.95	$-$ 0.05	1.0075	$-$ 0.0751
$y_0$	0.64	0.35	0.6337	0.3538
$z_0$	0.65	0.40	0.6606	0.4062
$\phi _x$	0.1667 $\pi$	$-$ 0.125 $\pi$	0.1625 $\pi$	$-$ 0.0060 $\pi$
$\phi _y$	0.125 $\pi$	0	0.1320 $\pi$	0.8750 $\pi$
$\phi _z$	0	0.5 $\pi$	0.02 $\pi$	0.01 $\pi$
*a*	0.29	0.33	0.4043	0.3320
*b*	0.29	0.29	0.2831	0.3340

**Fig. 12:**
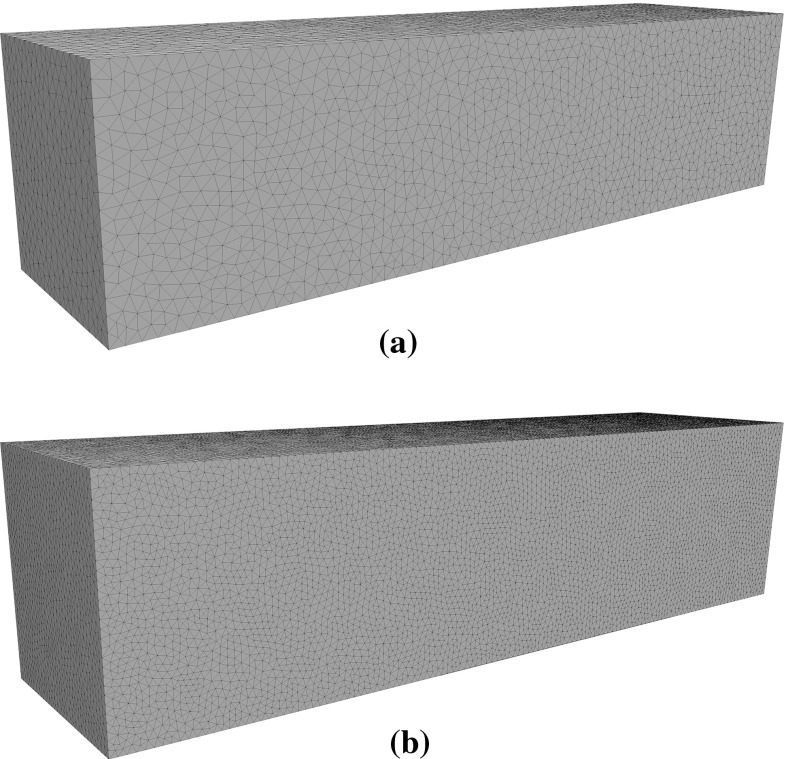
Meshes used for the three point bending problem. **a** Coarse mesh for the solution of the forward problem and **b** fine mesh for simulating measurements

The beam is meshed with an unstructured mesh consisting of 68,439 tetrahedral elements and 14,039 nodes. For simulating measurements a finer mesh consisting of 491,244 tetrahedral elements and 89,757 nodes is used. Both meshes used are illustrated in Fig. 12 while the locations of the cracks are shown in Fig. 14.

**Fig. 13:**
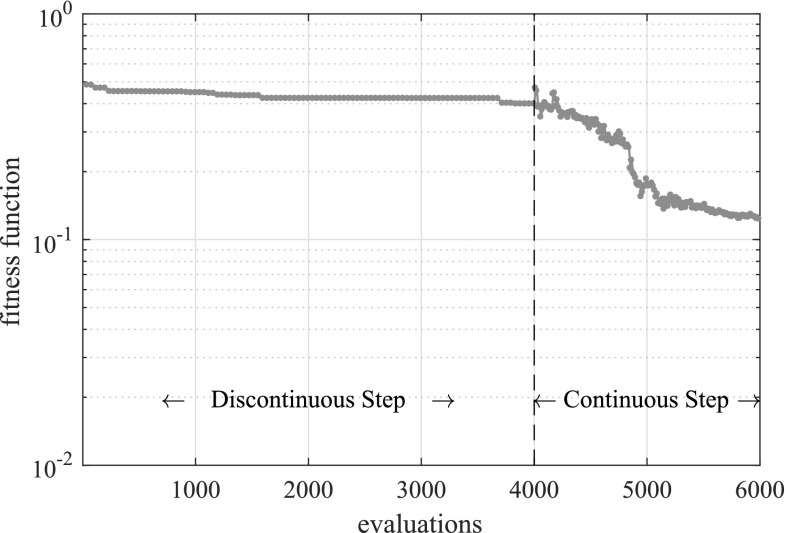
Convergence of the proposed multiscale strategy for the problem of a beam under three point bending

**Fig. 14:**
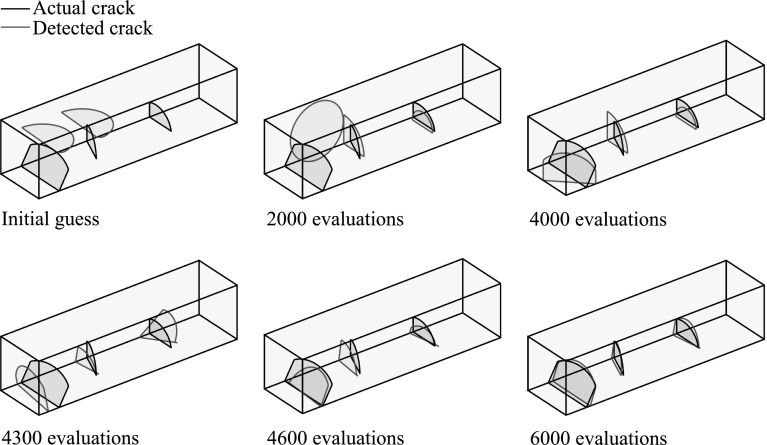
Best candidate solution after different numbers of evaluations for the problem of beam under three point bending

The limits for the parameters used in the first step of the optimization procedure were set to $x_{0\mathrm{min}}=-0.3$ units, $x_{0\mathrm{max}}=0.3$ unit, $y_{0\mathrm{min}}=-0.075$ units, $y_{0\mathrm{max}}=0.075$ unit, $z_{0\mathrm{min}}=-0.075$ units, $z_{0\mathrm{max}}=0.075$ unit, $a_{\mathrm{min}}=b_{\mathrm{min}}= 0.04$ units and $a_{\mathrm{max}}=b_{\mathrm{max}}= 0.08$ units. For the representation of parameters $x_0$, $y_0$ and $z_0$, 4, 2 and 2 binary digits were used respectively, two (4 possible values) for the angles defining the plane of the ellipse and one for the ellipse parameter. The population was set to 40 individuals and the mutation rate to 0.4 as in the previous example, however the the optimization was set to run for 4000 evaluations of the fitness function due to the increased complexity.

For the second part of the procedure the default parameters of the CMA-ES algorithm are adopted and the algorithm is set to run for 2000 evaluations of the forward problem. The maximum allowed difference between the parameters of the ellipse is set to $d_a=0.04$ units.

The fitness function value achieved by the best individual of the population is given as a function of the number of evaluations of the fitness function in Fig. 13, while in Fig. 14 the best solution after different numbers of evaluations is shown.

As illustrated in Fig. 14, a quite accurate fit can be achieved for all three cracks, nevertheless the number of evaluations required (6000) would be prohibitive for larger models. In addition, an increased number of evaluations was required in the first step compared to the previous example due to the increased number of cracks. However, the number of cracks would not be known in the general case, therefore a large number of evaluations (probably larger than the one used herein) might be necessary.

### Detection of two edge cracks in a wind turbine blade

In the last example a more complicated geometry is used to test the proposed scheme. More specifically, the geometry of a wind turbine blade with two edge cracks is considered. It should be noted that the example is only of academic interest since several simplifications are made which render the problem quite unrealistic. The most important of those simplifications are the following:

- A uniform material is considered for the whole blade. In reality the blade is hollow and made of a composite material, whose modeling complexity lies beyond the scope of this initial investigation.
- Static loading is considered.
- The crack locations considered are not consistent with the ones observed in actual turbine blades.

**Fig. 15:**
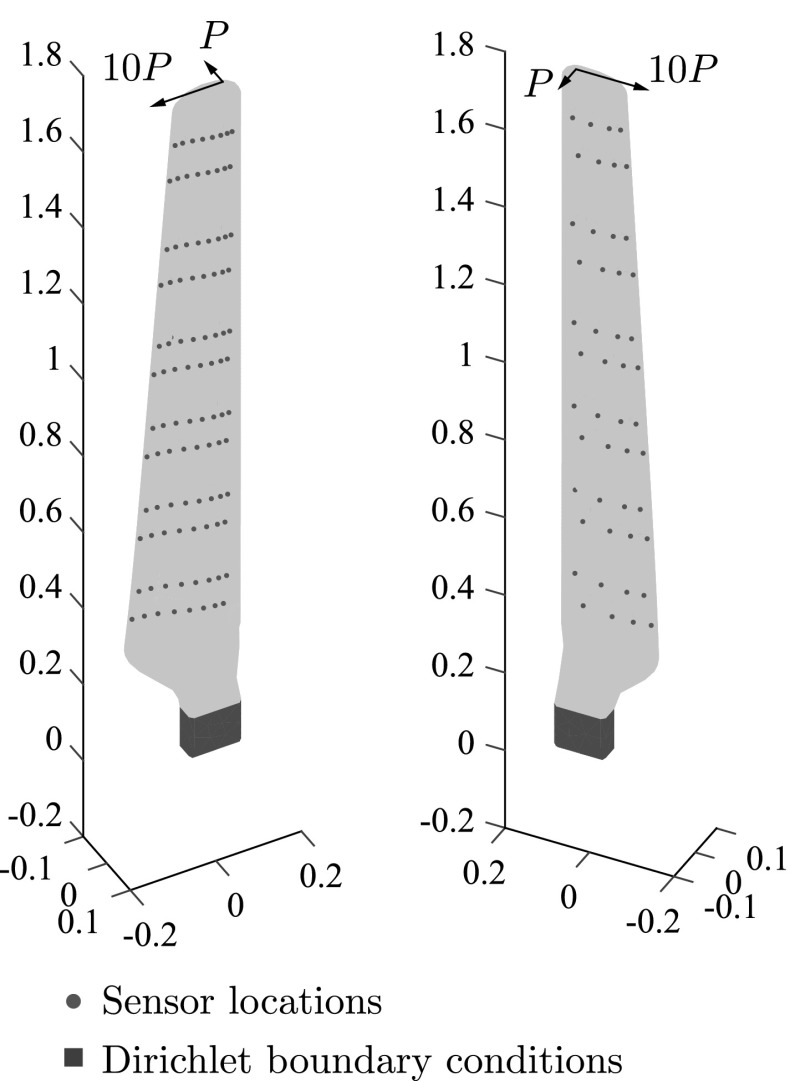
Wind turbine blade geometry, sensor locations and boundary conditions

**Fig. 16:**
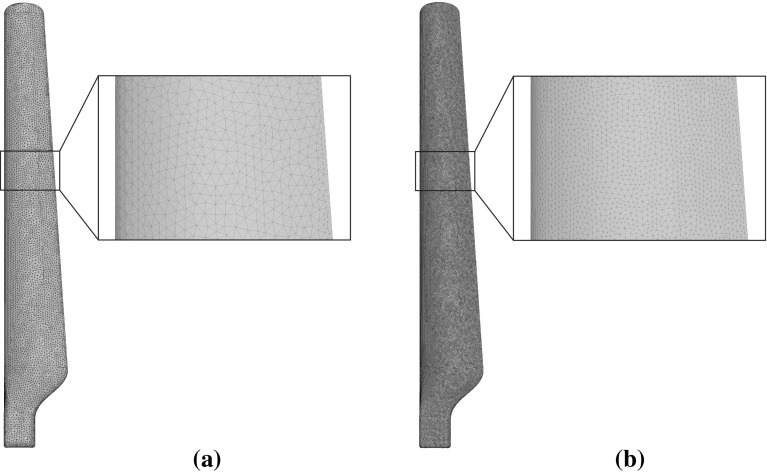
Meshes used for the wind turbine blade problem. **a** Coarse mesh for the solution of forward problems and **b** fine mesh for simulating measurements

In Fig. 15 the geometry of the blade as well as the sensor locations and applied boundary conditions are illustrated. Sensors are placed following the geometry of the blade, moreover one end of the blade is considered fixed (Fig. 15) while a uniform pressure $P=10$ is applied in a small area on the edge of the other end (Fig. 15). Academic material properties $E=200{,}000$ units and $\nu =0.3$ units were used.

Two unstructured meshes were used for the problem, a fine mesh for the simulation of the measured response of the blade consisting of 1,154,327 linear tetrahedral elements and 212,325 nodes (Fig. 16b), and a coarser mesh for the solution of forward problems consisting of 174,580 elements and 36,325 nodes (Fig. 16a).

**Fig. 17:**
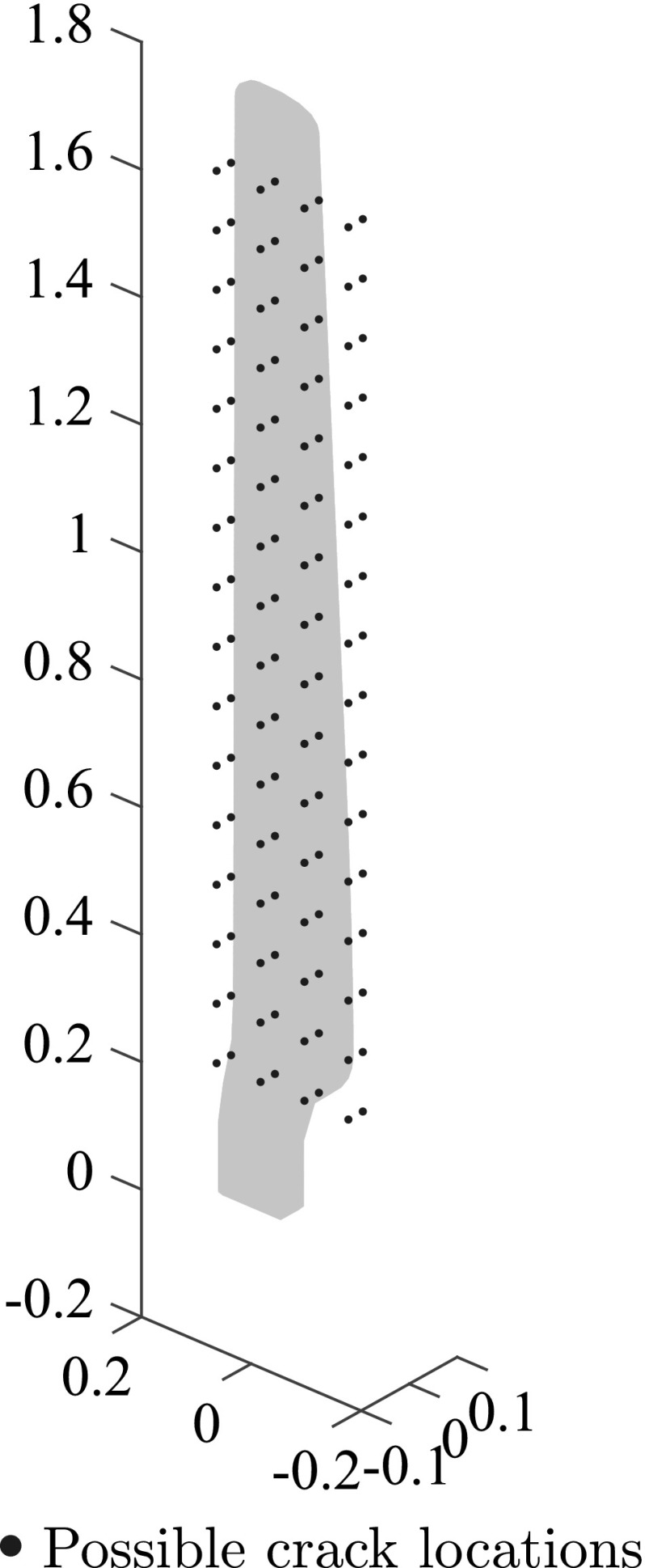
Possible crack locations for the first step of the multiscale crack detection scheme for the case of a wind turbine blade

The limits for the parameters used in the first step of the multiscale scheme of Sect. 5.3 were set to $x_{0\mathrm{min}}=-0.035$ units, $x_{0\mathrm{max}}=0.05$ unit, $y_{0\mathrm{min}}=-0.12$ units, $y_{0\mathrm{max}}=0.12$ unit, $z_{0\mathrm{min}}=0.2$ units, $z_{0\mathrm{max}}=1.6$ unit, $a_{\mathrm{min}}=b_{\mathrm{min}}= 0.08$ units and $a_{\mathrm{max}}=b_{\mathrm{max}}= 0.20$ units. For the representation of parameters $x_0$, $y_0$, $z_0$ 1, 2 and 4 binary digits (2, 4 and 16 possible values) were used respectively while two digits (4 possible values) were used for the rest of the parameters. In Fig. 17 all possible crack locations resulting from the above parameters are depicted. As in the previous example the possible number of cracks was set to four, the population size was set to 40 individuals, the mutation rate was set to 0.4 and the optimization was set to run for 2000 evaluations of the fitness function.

The default parameters of the CMA-ES algorithm are again adopted for the second part of the procedure. The maximum allowed difference between the two parameters of the ellipse is set to $d_a=0.10$ units.

**Fig. 18:**
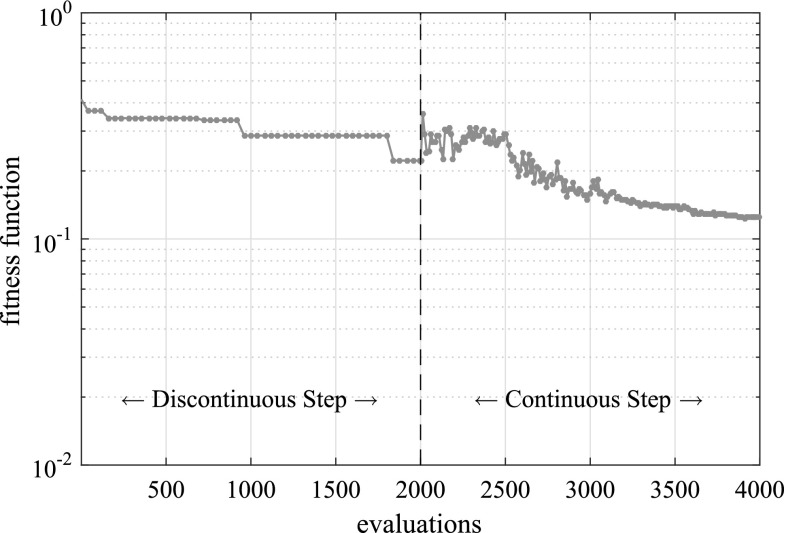
Convergence of the proposed multiscale strategy for the problem of a wind turbine blade with multiple cracks

**Fig. 19:**
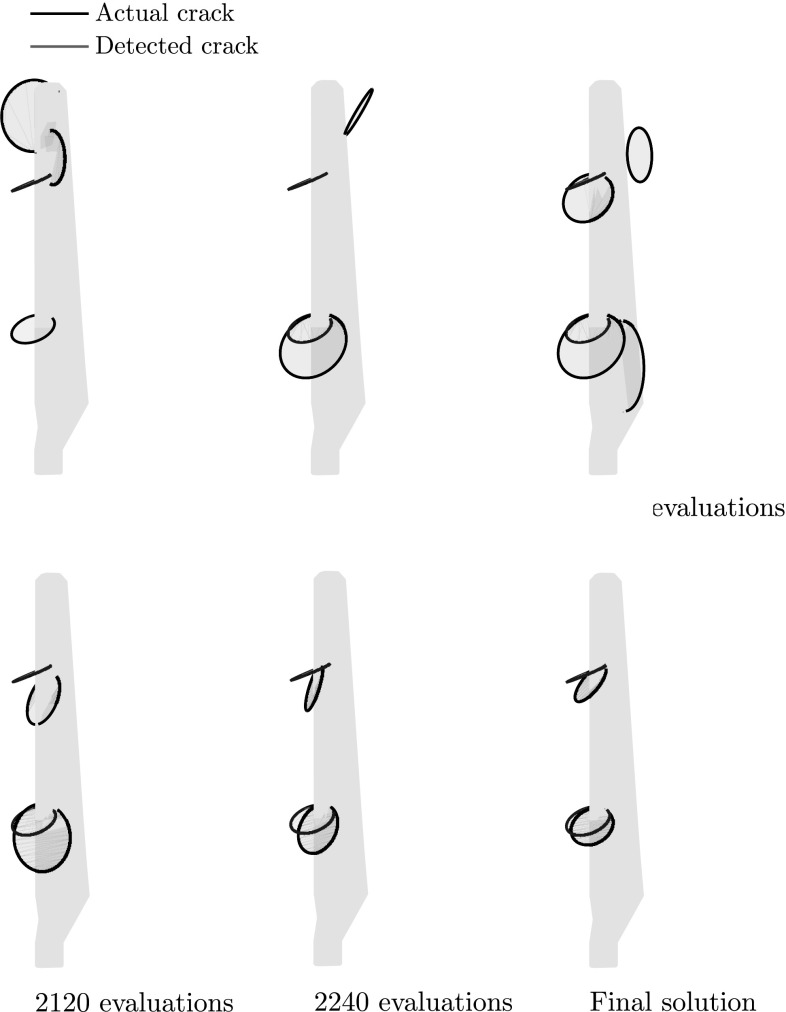
Best candidate solution after different numbers of evaluations for the problem of a wind turbine blade with two edge cracks

**Fig. 20:**
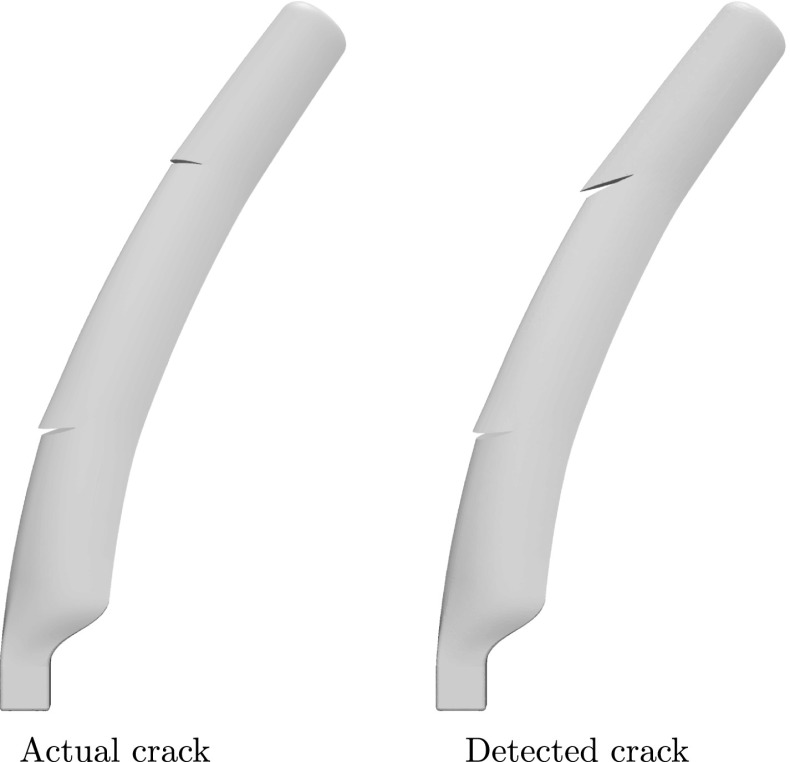
Deformed shape of the blade with the actual (left) and detected cracks (right)

In Fig. 18 the fitness function value achieved by the best individual of the population is given as a function of the number of evaluations of the fitness function, while in Fig. 19 the optimal solution after successive evaluations is illustrated. It should be noted that because of the more complicated geometry, the whole ellipses are plotted rather than only the parts of the ellipses that lie within the structure as in previous examples. In Fig. 20 the deformed shape of the blade with the actual and predicted cracks is plotted and in Fig. 20 the deformed shape of the blade is given with the actual and the detected cracks. Although the accuracy is decreased compared to the previous examples, the number of cracks and rough locations can still be obtained. This reduced accuracy can be attributed mostly to the fact that the applied loading does not activate both cracks equally making it harder to accurately detect the upper crack.

**Fig. 21:**
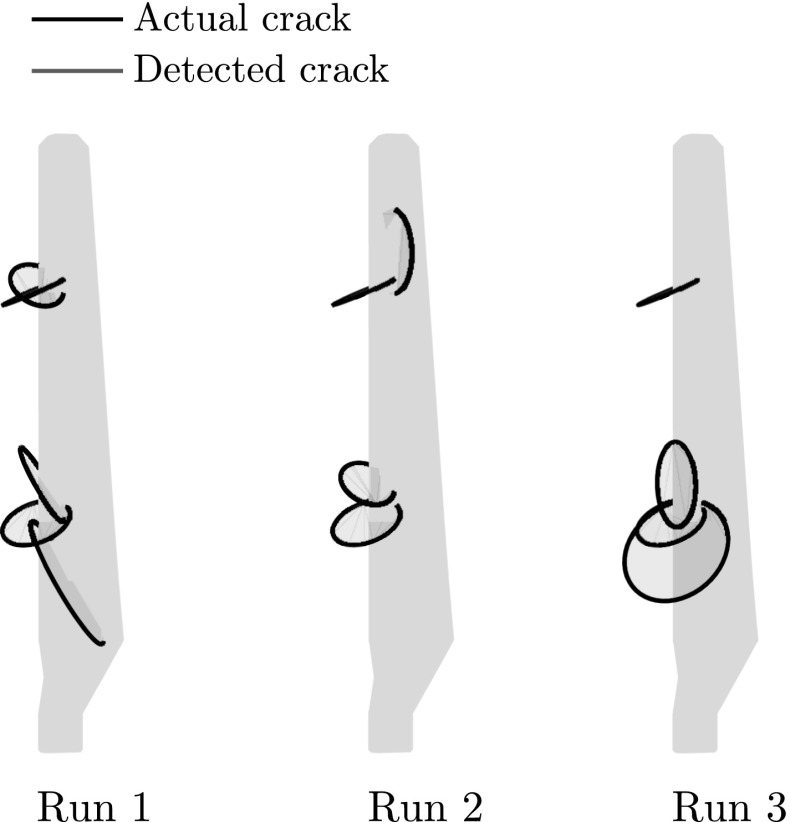
Best candidate from the first step of the solution for three alternative runs

It should be remarked that due to the increased complexity of the present problem and the stochastic nature of the optimization procedure it is not always possible to detect both of the cracks, this is illustrated in Fig. 21 where the best candidate obtained at the first step of the procedure is given for alternative runs. More specifically, in the first case (Run 1) both cracks are detected while in the following two cases (Run 2 and Run 3) only one of the cracks is accurately detected. The second run in particular is of special interest since one of the detected cracks (the upper crack) would result in zero or negative crack opening displacements and therefore would be physically meaningless. In the present version of the method no particular care was taken for those cases, however in future works those cases can be dealt with either by locating and penalizing those cracks or by including contact which would prevent negative crack openings.

## Conclusions

A methodology for the detection of multiple cracks in 3D solids of arbitrary geometries was presented, resulting via fusion of a recently introduced XFEM variant [55, 56] with a multiscale optimization strategy. The latter comprises a discrete step, where genetic algorithms are employed, and a continuous step employing the CMA-ES algorithm [57].

The method was tested in numerical examples involving the detection of multiple cracks in solids of non-regular geometries and promising results were obtained. Nevertheless, before the method may be implemented onto practical problems several improvements have been identified as future work, for alleviating certain methodological limitations. More specifically:

- The computational cost associated with the solution of the forward problems is high, which in turn increases the total computational cost since those problems need to be solved thousands of times. This could be a prohibiting factor for several applications, thus special techniques, such as model order reduction [24, 25, 82], would be required to extend the method’s applicability.
- In some cases the cracks detected by the method are physically meaningless since they involve zero or negative crack opening displacements. This can be dealt with by detecting and penalizing those cracks or by including contact in the model of the forward problem.
- Despite adoption of a rather high number of sensors, the inverse problem may result as ill-conditioned, especially for the more complex geometry and loading conditions of the third problem tested. A possible remedy to this problem could result via use of multiple loading cases, as in the work of Rabinovich et al. [41]. Such a remedy would only be possible once the size of the forward problems has been reduced, as mentioned above, since it would further increase the total number of evaluations of the forward problem.

The proposed method offers a highly promising tool towards the accurate detection of multiple cracks in complex engineered systems, simulated in the three dimensional domain.
